# [Corrigendum] 17-AAG synergizes with Belinostat to exhibit a negative effect on the proliferation and invasion of MDA-MB-231 breast cancer cells

**DOI:** 10.3892/or.2026.9142

**Published:** 2026-05-27

**Authors:** Yu Zuo, Heng Xu, Zhifeng Chen, Fengmin Xiong, Bei Zhang, Kaixian Chen, Hualiang Jiang, Cheng Luo, Hao Zhang

Oncol Rep 43: 1928–1944, 2020; DOI: 10.3892/or.2020.7563

Subsequently to the publication of the above paper, an interested reader drew to the authors' attention that, concerning the Transwell migration assay experiments shown in Fig. 6F on p. 1940, the ‘NC’ and ‘siTEAD3’ experiments showed an overlapping section of data, such that data which were intended to show the results from differently performed experiments had apparently been derived from the same original source. The authors proposed removing Fig. 6F from the figure, as they considered that the conclusions of these experiments were sufficiently well supported by the data shown in Fig. 6D and E, although the Editor expressed to them our preference that the authors should repeat these experiments, if necessary, to rectify the original errors made during the inaccurate assembly of data in Fig. 6F.

The authors were willing to perform the requested experiments, and the revised version of Fig. 6, showing the replacement data for the migration assay experiments in Fig. 6F, is shown on the next page. The authors wish to draw the readers' attention to the fact that the statistical significance of the experiments featured in the new Fig. 6F was not exactly the same as that in the original figure (note the change to the wording of the figure legend opposite, which is highlighted in bold); however, the conclusion that knocking out TEAD family proteins significantly inhibits the migration of MDA-MB-231 cells is completely consistent with the conclusion of the original article. This difference in the statistical significance of the results is reflected in a proposed change of the wording to describe the results of Fig. 6F in the manuscript; therefore, the final sentence of the Results section, left-hand column on p. 1941, should now read as follows: ‘As shown in the results of the cell migration assay (Fig. 6F), **knockdown of TEAD family proteins inhibits the migration of MDA-MB-231 cells**’.

The authors regret the errors that were made during the preparation of the Fig. 6. They are grateful to the editor of *Oncology Reports* for allowing them the opportunity to publish this Corrigendum, and all the authors agree to this publication. Furthermore, they apologize to the readership for any inconvenience caused.

## Figures and Tables

**Figure 6.**
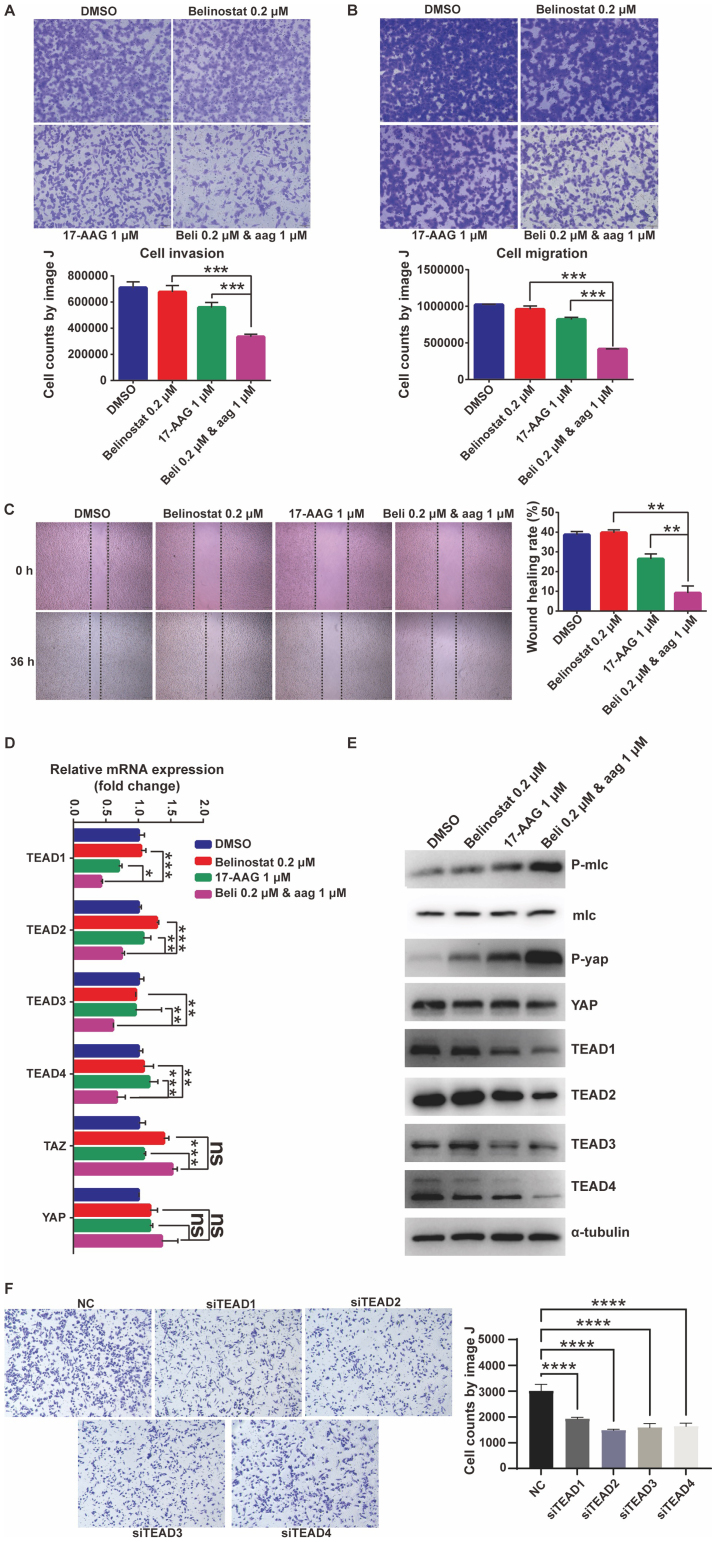
Enhanced inhibition of the migration and invasion of MDA-MB-231 cells. (A) Transwell migration assays demonstrated that enhanced inhibition was observed for the combination group of 17-AAG and Belinostat in MDA-MB-231 cells. Magnification, ×40. (B) Transwell invasion assay demonstrated that enhanced inhibition was observed for the combination group of 17-AAG and Belinostat in MDA-MB-231 cells. Magnification, ×40. (C) Wound healing assays revealed that enhanced inhibition was observed for the combination group of 17-AAG and Belinostat in MDA-MB-231 cells. Magnification, ×40. (D) Significant downregulation of expression was observed for the TEAD family proteins in the combination group compared with in the treatment groups of 17-AAG or Belinostat alone. (E) Phosphorylation of YAP and MLC was significantly increased in the combination group, indicating the suppression of migration and invasion-associated pathways. Decreased protein abundance of the TEAD family was consistent with the mRNA downregulation. (F) Transwell migration assays demonstrated that the knockdown ofTEADs genes inhibited the migration of MDA-MB-231 cells. ****P<0.0001. Magnification, ×40. Data are presented as the mean ± SEM. *P<0.05, **P<0.01, ***P<0.001. p-,phosphorylated; TEAD, TEA domain family member; ns, not significant; YAP, YY1 associated protein 1; TAZ, tafazzin; MLC, modulator of VRAC current 1; si, small interfering RNA; NC, negative control.

